# A two-arm analysis of the immune response to heterologous boosting of inactivated SARS-CoV-2 vaccines

**DOI:** 10.1038/s41598-023-46053-8

**Published:** 2023-10-31

**Authors:** Arnone Nithichanon, Ludthawun Kamuthachad, Kanin Salao, Wisitsak Phoksawat, Chatcharin Kamsom, Surasakdi Wongratanacheewin, Chonlatip Pipattanaboon, Sakawrat Kanthawong, Umaporn Yordpratum, Sirinart Aromseree, Atibordee Meesing, Piroon Mootsikapun, Steven W. Edwards, Supranee Phanthanawiboon

**Affiliations:** 1 Department of Microbiology, Faculty of Medicine, Khon Kaen University, Khon Kaen, Thailand; 2 Research and Diagnostic Center for Emerging Infectious Diseases (RCEID), Khon Kaen University, Khon Kaen, Thailand; 3 Institute of Infection, Veterinary and Ecological Sciences, University of Liverpool, Liverpool, UK; 4 Infectious Disease Unit, Department of Medicine, Faculty of Medicine, Khon Kaen University, Khon Kaen, Thailand

**Keywords:** Immunology, Microbiology, Vaccines

## Abstract

Several vaccine programs were introduced during the COVID-19 pandemic, which included inactivated virus, DNA viral vectors and mRNA vaccines. Booster programs are recommended, especially for those in high-risk groups. However, many of these booster programs involve heterologous vaccines. This study enrolled volunteers who first received two full-dose CoronaVac vaccinations before receiving heterologous boosters with DNA- and/or mRNA-vaccines for an additional 2 doses (n = 40) or an additional 3 doses (n = 16). Our results showed no difference in side effects, neutralizing antibodies, or T-cell responses for any of the heterologous vaccination programs. However, the neutralizing capacity and IFN-γ responses against the Omicron variant in volunteers who received 4 or 5 doses were improved. Polarization of peripheral memory T cells after stimulation in all booster groups with Omicron peptide showed an increased trend of naïve and central memory phenotypes of both CD4+ and CD8+ T cells, suggesting that exposure to Omicron antigens will drive T cells into a lymphoid resident T cell phenotype. Our data support a continuous vaccination program to maximize the effectiveness of immunity, especially in people at high risk. Furthermore, the number of boosting doses is important for maintaining immunity.

## Introduction

The SARS-CoV-2 pandemic triggered the development of a range of vaccines of different designs and with varying efficacies of protection against infection^1^. The advent of these vaccines has had a major impact in preventing severe disease associated with systemic inflammation and chronic lung disease following infection, however, the fast waning of immune responses is a major concern for continued protection^2,3^. Andrew et al. showed that after primary vaccination with DNA- or mRNA-vaccines there was reduced effectiveness of immunity at 20 weeks compared 2–4 weeks^2^. This decreased vaccine effectiveness may be attributed to both waning immunity and the impact of circulating variants of concern (VOC) with altered immunogenicity^4^. The rate of decreased immunity is likely to be contingent upon factors such as the target population, type and number of vaccines received^5^. Because vaccine protection decreases over time, especially in the circulating levels of neutralizing antibodies^6,7^, booster vaccinations are recommended for sustained protection. There is current debate about the frequency and the need for continuous booster vaccinations in the general population, but it is recognised that high-risk groups (e.g. the elderly and those with underlying health conditions) may continue to benefit from booster programs^8,9^.

Many types of SARS-CoV-2 vaccines have been developed, the major types including whole inactivated virus, recombinant proteins, mRNA-, and viral DNA-vector vaccines, which have different efficacies against the original strain and for emerging variants^10,11^. Therefore, it is important to determine the optimal regimens for booster strategies, in particular when using heterologous vaccination schedules, in order to optimise effectiveness, particularly concerning the availability of different vaccines in countries across the globe^12^.

In the early stages of the pandemic, the vaccination strategy in many countries was dictated by the availability of particular vaccines. Many countries, especially Asian countries, used CoronaVac (CV) for their initial immunisation program^13,14^, but its efficacy in protection against infection, particularly against new variants, has been questioned^15^. Therefore, many countries are promoting booster program using other types of vaccines. It is therefore important to evaluate the effectiveness of these heterologous boosting programs and to establish the immune mechanisms that are stimulated, because it is recognized that boosting still only provides temporary protection against infection (especially mild disease)^16,17^. Boosting programs stimulate circulating antibody levels, but these may have varying neutralizing capacity against current variants circulating in the population^18,19^. The effectiveness of these strategies on the numbers and function of memory cells, however, is less well defined, and whether these play a role in limiting the more aggressive inflammatory activation mechanisms that underpin the morbidity and mortality associated with severe SARS-CoV-2 disease remains to be investigated.

In the present report, we describe both antibody and memory T cell subset responses in two cohorts of healthcare workers who received two initial vaccinations with CV, followed by 2 or 3 heterologous boosters with either mRNA- or DNA-vaccines. We show that both DNA- and mRNA-booster vaccines give rise to increased levels of circulating neutralizing antibodies and IFN-**γ** production after activation with SAR-CoV-2 proteins, but little change in memory T cells. These findings provide data to support the view that booster vaccinations should continue to maximize the effectiveness of immunity, especially for people at high risk of developing severe disease following infection.

## Methods

### Ethics statement and study participants

This study was approved by the Khon Kaen University Ethics Committee for Human Research (KKUEC, approval number HE641338). The experiments were performed in accordance with relevant guidelines and regulations. All participants provided written informed consent before recruitment to the study. This study focused on the type of COVID-19 vaccine and did not consider the specific manufacturers which producing these vaccines. All participants were followed-up from our previous study^20^ who were vaccinated with two doses of the CoronaVac vaccine and subsequently received another 2 or 3 booster DNA- or mRNA-vaccines. Eligibility criteria for those participating in the study were healthcare staff within Khon Kaen province aged > 18 years with no history of seizures, acute febrile illness, pregnancy, HIV infection, or immunomodulator agent usage.

### Blood sample collection and processing

Whole blood samples were collected in heparinized tubes (BD Biosciences, San Jose, USA) for isolation of peripheral blood mononuclear cells (PBMCs) by density gradient centrifugation using Lymphoprep and SepMate tubes (STEMCELL Technologies, USA) according to the manufacturer’s instructions. Isolated PBMCs were resuspended in R10 complete medium (RPMI-1640 supplemented with 10% fetal bovine serum (FBS), 100 U/mL penicillin–streptomycin (all from Gibco, Thermo Fisher Scientific, Waltham, USA) for subsequent experiments. EDTA- (BD Biosciences, San Jose, USA) blood samples were collected for complete blood count (CBC). Blood was also collected in clot gel tubes (BD Biosciences, San Jose, USA) to prepare serum by centrifugation at 500×*g* for 10 min and stored at − 20 °C for later analysis.

### Surrogate SARS-CoV-2 neutralization antibody detection

Neutralization antibodies (NT) were detected by a SARS-CoV-2 neutralization antibody detection kit (GenScript Biotech, NJ, USA) according to the manufacturer’s instruction. Briefly, serum samples as well as negative and positive controls assay were diluted 1:10 in sample dilution buffer and pre-incubated with horseradish peroxidase (HRP) conjugated SARS-CoV-2 receptor binding domain (RBD) of SARS-CoV-2 wild type strain (Wuhan Hu-1) or B.1.1.529 (Omicron) variant (GenScript Biotech, NJ, USA) at 37 °C for 30 min. The mixture was then transferred to a 96-well plate coated with human ACE2 protein (hACE2) and incubated at 37 °C for 15 min. After incubation, the supernatant was removed and washed 4 times with wash solution buffer. Tetramethylbenzidine substrate (TMB) was added and incubated at RT for 15 min followed by adding stop solution. The plate was read at 450 nm in a microtiter plate reader immediately afterwards. Percentage inhibition for each sample was calculated using the following formula:

$$\mathrm{\% Inhibition}=\left(1-\frac{\mathrm{OD\, value \,of \,sample}}{\mathrm{OD\, value\, of \,negative \,control }}\right)\times 100\mathrm{\%}.$$

Serum samples exhibiting an inhibition percentage of at least 30% were classified as positive for the presence of neutralizing antibodies, whereas those displaying less than 30% inhibition were considered as negative, based on the manufacturer’s guidelines.

### IFN-γ releasing assay by QuantiFERON

SARS-CoV-2 specific T cell responses were evaluated by IFN-γ release using a QuantiFERON SARS-CoV-2 kit (QIAGEN, Germantown, USA) in accordance with the manufacturer’s instructions. Briefly, 1 mL of heparinized blood was transferred into each QuantiFERON tube containing the SARS-CoV-2 RBD or S1S2 protein. In addition, blood samples were added to a Nil or Mitogen tube, which served as the negative and positive control, respectively. Samples were then incubated for 24 h at 37 °C before centrifugation at 500×*g* for 10 min to separate plasma. IFN-γ (IU/mL) in plasma samples was then measured by an enzyme-linked immunosorbent assay (ELISA) using a DS2 instrument (QIAGEN, Germantown, USA).

### Determination of memory T cell subsets

To evaluate T cell activation, freshly isolated PBMCs (1 × 10^6^ cells/well) were activated with SARS-CoV-2 Omicron BA.1 (S1) peptides at a final concentration of 2 µg/mL in R10 medium at 37 °C with 5% CO_2_ for 20 h. After incubation, cells were harvested and stained with the following monoclonal antibodies: anti-human CD3-APC-Cy7; anti-human CD4-V450 (Thermo Fisher Scientific, Waltham, USA); anti-human CD8-FITC; anti-human CD62L-APC (BD Pharmingen, San Diego, CA, USA); anti-human CD45RA-PE-Cy7 for 30 min at 4 °C in the dark. All samples were analysed with a BD FACSLyric Flow Cytometer (BD Biosciences, San Jose, CA, USA) and analyzed by FlowJo Software version 10 (Flowjo, LLC, Ashland OR, USA). The absolute numbers of lymphocytes and T lymphocyte subsets were calculated from complete blood count data obtained from the Associated Medical Science (AMS) Wellness Center Khon Kaen University.

### Statistical analysis

Statistical analyses were performed using GraphPad Prism Software version 9.5.1 (GraphPad Software, Inc., San Diego, CA, USA). Normal distribution of data was checked with D’Agostino & Pearson test. A comparison of non-normal distributed data was performed with Kruskal–Wallis test with Dunn’s multiple comparisons test. Comparison of paired samples was performed using a two-tailed paired t test. Chi-square was applied to test for differences of categorical data. Correlation of numerical data was performed using linear regression.

## Results

### Heterologous vaccination-induced virus-inhibiting antibodies

**Figure 1:**
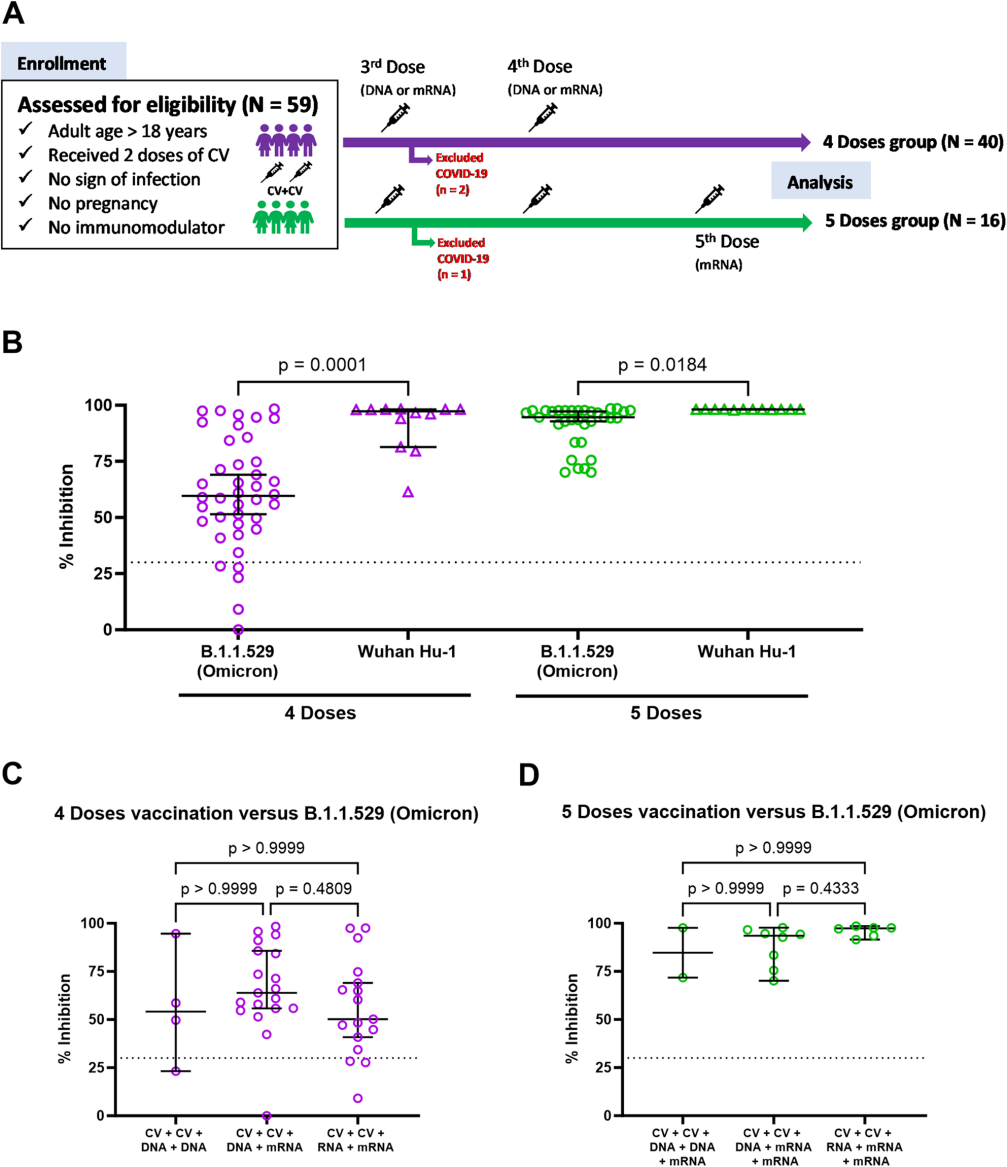
Comparison of plasma inhibitory efficacy against B1.1.529 (Omicron) and Wuhan Hu-1 following different vaccination strategies. Serum samples of participants with 4 doses (n = 40) or 5 doses (n = 16) were collected to evaluate % inhibition against B1.1.529 (Omicron) and Wuhan Hu-1 SARS-CoV-2. **(A)** Shows a flow of participants through the study. In (**B–D**) data represent individual participant measurements (open purple circle for 4 doses, open green circle for 5 doses) with the horizontal lines showing median and 95% confidence interval (95% CI) and dash line represents a positive cut-off. **(B)** Shows differences in % inhibition of different viral strains and different vaccinations regimens with responses against Omicron (open purple circle, open green circle) and against Wuhan Hu-1 (open purple triangle, open green triangle). **(C,D)** Show % inhibition of B1.1.529 (Omicron) with different vaccination strategies after 4 doses or 5 doses, respectively. Data were analysed using Kruskal–Wallis test with Dunn’s multiple comparisons test. Statistical significance was determined at a *p*-value < 0.05.

This was a follow-on study from a cohort of volunteers who originally received two doses of CV^20^ and who subsequently received either another 2 (n = 40) or 3 (n = 16) heterologous boosters. The demographics of these participants are shown in Table 1. Both cohorts had similar age profiles and similar ranges of mild side effects, that were mainly fatigue, headache and fever. There were also no differences in the underlying conditions of the participants but a slight difference in the sex of the participants, with a higher proportion of females who received 4 doses. The booster schedules were as follows: 6 received two DNA vaccines; 23 received two mRNA vaccines; 27 received DNA-prior mRNA-vaccines. All 16 who received 5 doses had an mRNA vaccine for their 5th vaccination (Table 1, Fig. 1A). The time interval (in days) between blood sample collection after the 4th dose and the 5th dose was significantly different (*p* < 0.0001). This sampling interval was much shorter after the 5th dose of vaccine (Supplementary Table 1) and while there was a longer period between the 1st and 2nd CV vaccination in the cohort who received five doses, both groups were sampled at similar times after the 3rd and 4th doses. Around 12–17% of the participants reported antigen positive COVID-19 infection during the course of this study, a similar proportion in each cohort.

**Table 1:** General demographic data of participants in this study.

Parameter	4 Doses (N = 40)	5 Doses (N = 16)	*p*-value
Average age in years (range)	45.2 (24–69)	42.9 (27–55)	0.5223
Female, n (%)	27 (67.5%)	6 (37.5%)	0.0393*
Underlying condition, n (%)			
Hypertension	4 (10.0%)	1 (6.3%)	0.6566
Allergy or asthma	4 (10.0%)	1 (6.3%)	0.6566
Dyslipidemia	3 (7.5%)	1 (6.3%)	0.8697
Diabetes mellitus	3 (7.5%)	0 (0.0%)	0.2602
Hyperthyroid	2 (5.0%)	2 (12.5%)	0.3249
Chronic hepatitis B	1 (2.5%)	0 (0.0%)	0.5234
Breast cancer	1 (2.5%)	0 (0.0%)	0.5234
Syringomyelia	1 (2.5%)	0 (0.0%)	0.5234
Systemic lupus erythematous	1 (2.5%)	0 (0.0%)	0.5234
Vaccination program, n (%)			
CV + CV + DNA + DNA (± mRNA^a^)	4 (10.0%)	2 (12.5%)	0.7847
CV + CV + DNA + mRNA (± mRNA^a^)	19 (47.5%)	8 (50.0%)	0.8657
CV + CV + mRNA + mRNA (± mRNA^a^)	17 (42.5%)	6 (37.5%)	0.7312
Days after the last vaccination, median (interquartile range)	217 (204–219)	19 (8–39)	< 0.0001*
Side effects after vaccination, n (%)	16 (40.0%)	3 (18.8%)	0.1644
Fatigue	9 (56.3%)	1 (33.3%)	0.4657
Headache	8 (50.0%)	1 (33.3%)	0.5957
Fever	5 (31.3%)	2 (66.7%)	0.2432
Muscle pain	2 (12.5%)	1 (33.3%)	0.3638
Numbness	1 (6.3%)	0 (0.0%)	0.6564
Skin rash	1 (6.3%)	0 (0.0%)	0.6564
COVID-19 after the final dose, n (%)	7 (17.5%)	2 (12.5%)	0.6453

*Statistically significance.

^a^Only in the 5 Doses group had mRNA vaccination.

Peripheral blood samples were collected after either 4 or 5 doses of vaccine (Fig. 1A) and serum samples were first tested for surrogate neutralization against either B.1.1.592 (Omicron) and the wild type of Wuhan Hu-1 virus (Fig. 1B). Either vaccination regimen was very effective at neutralizing the Wuhan Hu-1 strain. There was more variation in the neutralizing effect of serum against Omicron strain after 4 doses with 5 participants show neutralizing capacity that was below the detection cut-off. Similarly, after 5 doses of vaccine, the ability of serum to neutralize Omicron showed more variation than against Wuhan Hu-1. There were no statistical differences in inhibition patterns amongst the cohorts who had different DNA- or mRNA-booster regimens after either 4 or 5 doses (Fig. 1C,D).

We then further analysed these data considering the time of blood sampling after the final (either 4th or 5th) dose of vaccine. For those who received 4 doses, most of these blood samples were collected around 200 days after the final vaccination (Supplementary Fig. 1A) and there was a wide range of neutralizing activity against Omicron in this cohort. In contrast, there was a significant correlation between the time of blood sampling and Omicron neutralizing activity in those who received 5 doses (*p* < 0.0001, r =  − 0.7369, Supplementary Fig. 1B).

### Heterologous vaccination induced IFN-γ production against SARS-CoV-2 antigens

**Figure 2:**
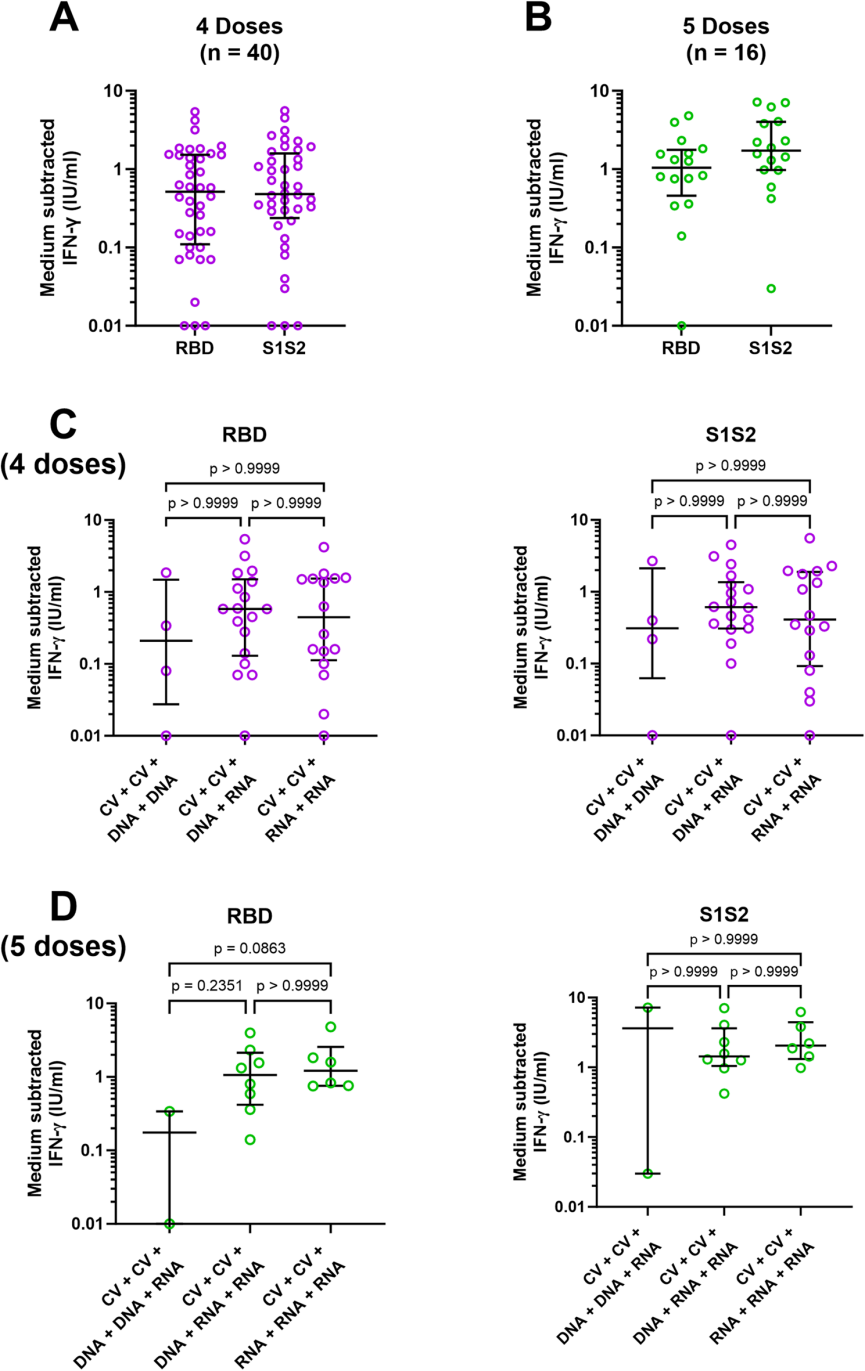
Specific T cell responses measured as IFN-γ release upon in vitro stimulation with SARS-CoV2 receptor-binding domain (RBD) or S1S2 peptides after four or five doses of vaccination. Peripheral blood mononuclear cells (PBMCs) from participants after 4 doses or 5 doses were stimulated with RBD or S1S2 peptides. IFN-γ release was quantified using QuantiFERON. Data are shown as individual dot plots after medium (control) subtracted IFN-γ levels (open purple circle for 4 doses, open green circle for 5 doses) with horizontal lines showing median and interquartile ranges. IFN-γ levels release by samples after 4 doses and 5 doses in response to RBD **(A)** or S1S2 peptides **(B)** were compared using Mann Whitney test. Levels of IFN-γ release by samples after different vaccine combinations of 4 doses **(C)** or 5 doses **(D)** were analysed using Kruskal–Wallis test with Dunn’s multiple comparisons test. Statistical significance was determined at a *p*-value < 0.05.

We then investigated the effects of vaccination boosting on other anti-viral immune mechanisms. First, we measured the ability of immune cells to generate IFN-γ after stimulation of PBMC with specific viral antigens. Heparinised peripheral venous blood samples were stimulated with SARS-CoV-2 RBD or S1S2 protein, alongside incubation in culture medium alone as a control, and IFN-γ production was quantified by QuantiFERON. Figure 2 shows that after 4 vaccine doses similar levels of IFN-γ were generated in response to RBD protein (median 0.52 IU/mL: IQR at 0.11–1.52 IU/mL) or S1S2 antigen (median 0.48 IU/mL: IQR at 0.24–1.59 IU/mL: Fig. 2A). Similarly, in those who received 5 vaccine doses, IFN-γ levels generated in response to RBD protein (median 1.05 IU/mL: IQR at 0.46–1.76 IU/mL) or S1S2 antigen (median 1.73 IU/mL: IQR at 0.97–4.01 IU/mL: Fig. 2B) were not significantly different. When these data were re-analysed according to the types of vaccine used for boosting, there were no significant differences between the groups irrespective of whether the participant had 4 or 5 doses (Fig. 2C,D).

There were no significant correlations between the levels of IFN-γ release and time of sample collection after 4 vaccination doses in response to either RBD or S1S2 (Supplementary Fig. 2A) or after 5 doses in response to RBD or S1S2 (Supplementary Fig. 2B). Similarly, there were no significant correlations between viral (Omicron) inhibition and IFN-γ levels after 4 vaccine doses, but a trend towards a positive correlation between IFN-γ production and viral inhibition after 5 doses that was statistically-significant after stimulation by S1S2 protein (*p* = 0.0204, Supplementary Fig. 3).

### Heterologous vaccination increased lymphoid resident memory T cells upon in vitro Omicron antigen re-stimulation

Isolated PBMCs were incubated with medium (control or resting) or immunogenic peptides derived from Omicron before surface marker staining for memory T cell subpopulations. Gating strategies identified CD4+ or CD8+ T cells as: naïve (CD45RA+/CD62L+); central memory (CM, CD45RA−/CD62L+); effector memory (EM, CD45RA−/CD62L−); effector memory with RA positive (EMRA, CD45RA+/CD62L−), and are shown in Supplementary Fig. S4. To avoid any interference from samples that had changes in the numbers of singlet cells after stimulation, we excluded from the analysis 3 samples from the 4 doses group that had changes in numbers of singlet cells of > 10% (Supplementary Fig. S5). When these 3 samples were excluded, there were no differences in numbers of lymphocytes recovered for analysis after stimulation.

**Figure 3:**
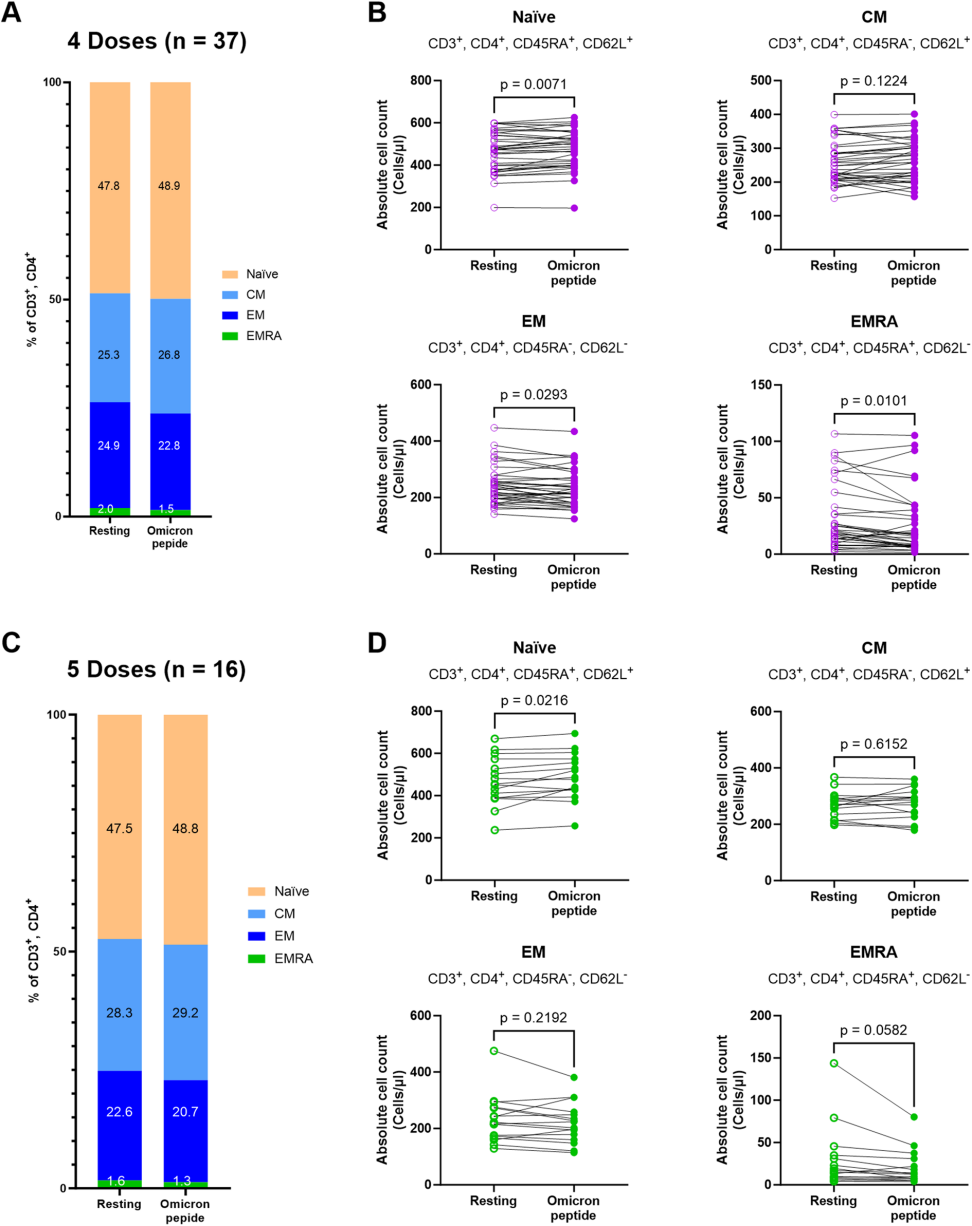
CD4+ T helper lymphocyte subset changes upon stimulation of PBMC from participants with B.1.1.529 (Omicron) peptide after 4 or 5 doses of heterologous vaccination. PBMCs from participants were cultured with medium control (Resting) or Omicron peptide prior to surface marker staining and flow cytometry analysis for CD4+ T helper lymphocytes. Average percentages of T helper lymphocytes with naïve, central memory (CM), effector memory (EM), and effector memory RA (EMRA) phenotypes from participants receiving 4 doses **(A)** or 5 doses **(C)** are shown. Absolute cell numbers of each phenotype were calculated and individually plotted, with lines connecting the same participant before and after stimulation (purple open circle; cell count in Resting samples from the 4th dose group, purple filled circle; cell count after stimulation of samples from the 4th dose group) **(B)**. The same analysis was performed on samples from the 5th dose group (green open circle; cell count in Resting, green filled circle; cell count after stimulation of samples from the 5th dose group) **(D)**. Statistically-significant changes in the paired samples were calculated with a paired t test, with significance determined at a *p*-value < 0.05.

Overall numbers of resting or Omicron peptide-stimulated memory T cell subpopulations were similar after 4 or 5 doses of vaccine. In more detail, the average percentages of each CD4+ memory T cell subpopulation in the 4 doses group are shown in Fig. 3A, while the populations of the 5 doses group are shown in Fig. 3C. We then normalized the data to calculate the absolute number of cells and found a statistically-significant increased number of naïve cells (*p* = 0.0071) but a decreased number of EM (*p* = 0.0293) and EMRA (*p* = 0.0101) after stimulation with Omicron peptides (Fig. 3B). In the cohort that received 5 doses, the absolute cell counts of each CD4+ memory T cell subpopulation revealed statistically-significant increased naïve cells (*p* = 0.0216) with decreased EMRA (*p* = 0.0582; Fig. 3D). We then determined whether there were any correlations in changes in the numbers of different CD4+ memory cell subpopulations. There were statistically-significant strong negative correlations (Supplementary Fig. S6) of CM and EM (*p* < 0.0001 and r =  − 0.7018 for 4 doses group; *p* < 0.0001 and r = − 0.6864 for 5 doses group), and CM and EMRA (*p* < 0.0001 and r =  − 0.3736 for 4 doses group; *p* = 0.0540 and r =  − 0.2401 for 5 doses group). There were also positive correlations between EMRA and EM cells after both 4 (*p* < 0.0001) and 5 doses (*p* = 0.0155).

**Figure 4:**
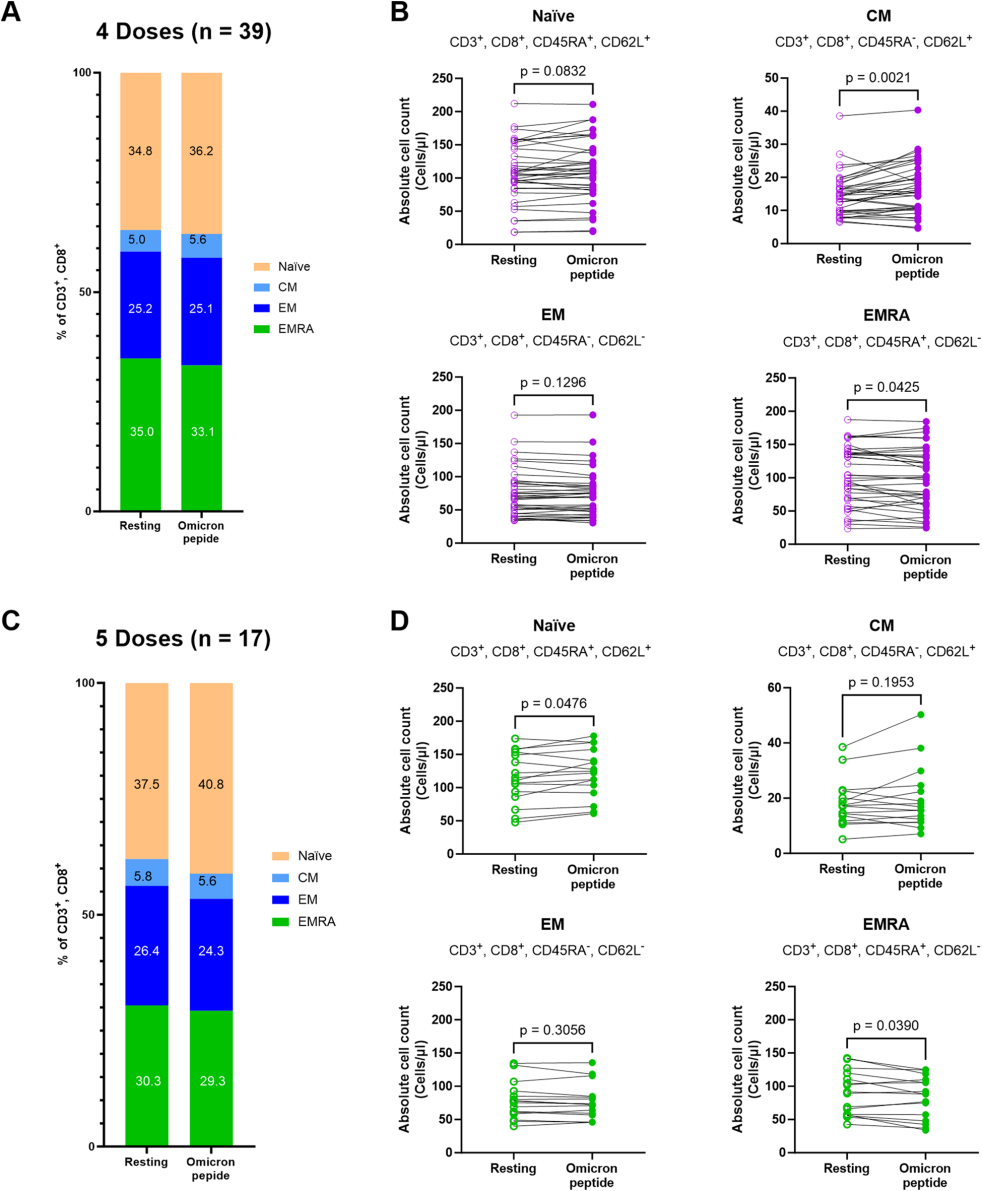
CD8+ cytotoxic T lymphocyte subset changes upon stimulation of PBMC from participants with B.1.1.529 (Omicron) peptide after 4 or 5 doses of heterologous vaccination. PBMCs from participants were cultured with medium control (Resting) or Omicron peptide prior to surface marker staining and flow cytometry analysis for CD8+ T cytotoxic lymphocytes. Average percentages of cytotoxic T lymphocytes with naïve, central memory (CM), effector memory (EM), and effector memory RA (EMRA) phenotypes from participants receiving 4 doses **(A)** or 5 doses **(C)** are shown. Absolute cell numbers of each phenotype were calculated and individually plotted, with lines connecting the same participant before and after stimulation (purple open circle; cell count in Resting samples from the 4th dose group, purple filled circle; cell count after stimulation of samples from the 4th dose group) **(B)**. The same analysis was performed on samples from the 5th dose group (green open circle; cell count in Resting, green open circle; cell count after stimulation of samples from the 5th dose group) **(D)**. Statistically-significant changes in the paired samples were calculated with a paired t test, with significance determined at a *p*-value < 0.05.

The average percentages of each CD8+ memory T cell subpopulation from the 4th and 5th doses groups are shown in Fig. 4A,C. Calculation of the absolute cell counts of each CD8+ memory T cell subpopulation revealed several trends, some of which were statistically-significant. Naïve and CM cells increased after stimulation (*p* = 0.0832 and 0.0021 in the 4th dose group; *p* = 0.0476 and 0.1953 in the 5th dose group, respectively) and decreased EMRA: *p* = 0.0301 in the 4th dose group; *p* = 0.0390 in the 5th dose group (Fig. 4B,D). There were statistically-significant negative correlations of CM and EM (*p* < 0.0001 and r =  − 0.3706 for the 4th dose group; *p* = 0.0287 and r = − 0.2982 for the 5th dose group), and CM versus EMRA (*p* < 0.0001 and r = − 0.4370 for the 4-dose group). CM and naïve phenotypes were positively correlated (Supplementary Fig. S7).

## Discussion

At the peak of the COVID-19 pandemic, many countries, particularly developing countries, were faced with limited availability of both the amounts and types of vaccines, which resulted in the introduction of vaccination strategies that included fractional dosing and heterologous boosting regimens. In many Asian countries, such as Thailand, initial vaccination was with two doses of CV, but as more vaccines became available, both DNA- and mRNA-based vaccines have been used in homologous and heterologous booster programs^21,22^. There is much interest in determining the long-term safety aspects of continuous vaccine boosting programs and their effectiveness in preventing serious conditions arising from SARS-CoV-2 infection^19,23^. In this study, we have followed participants who initially received two doses of CV^20^ and subsequently received another 2 doses of heterologous DNA- or mRNA-vaccines, and a cohort who then went on to have a 3rd booster of a mRNA vaccine.

The participants in our study were able to choose their type of vaccination program following their initial 2 doses of CV and had access to either DNA- or mRNA-vaccines for their 3rd and 4th doses: those who had 5 doses all chose mRNA vaccines. There were no significant differences in the reported side effects (mild) with any booster regimen and no differences in the rates of infection with SARS-CoV-2 (as determined by the antigen test), which were all reported as mild disease not requiring any medical interventions. This is similar to a study of heterologous vaccination with DNA- and mRNA-vaccines in healthcare workers in Germany, who also reported no differences in side effects compared to homologous vaccination^24^. Our data are also in agreement with a reported heterologous vaccine schedule in Northern Thai people in which protection against Omicron was reported at 31% after the third dose, rising to 75% after the fourth dose^25^. The rate of COVID-19 infection after the final boosting dose in our study was at 12–17%. A previous randomised trial in Hong Kong adults reported similar numbers of COVID-19 infections within 4–6 months after receiving a third dose including: 13/85 (15%) after 3 doses CV; 16/104 (15%) after 2 doses after CV priming prior to an mRNA booster; 16/96 (17%) after 2 doses of mRNA vaccine prior to CV boosting; 13/93 (14%) after 3 doses of mRNA vaccination. The infection rate after vaccination may be explained by a waning immunity and the impact of circulating variants of concern (VOC)^4^. A study of Brazilian people who received two doses of CV 6 months prior to boosting with DNA- or mRNA-vaccine suggested that the heterologous boosting resulted in more robust immune responses than homologous boosting and hence might enhance protection^26^. However, a systematic meta-analysis study reported that heterologous and homologous three-dose vaccination regimens were comparably effective in preventing COVID-19 infections^27^.

A randomised trial of heterologous prime-boost COVID-19 vaccine schedules across the UK (the Com-COV study) reported superior immunogenicity compared to homologous DNA vaccine prime-boost^28^. The immunogenicity measured after the third boosting dose also appeared to be more potent in another randomized trial (COV-BOOST)^29^. Moreover, heterologous boosting was reported to have superior efficacy to homologous boosting in participants with CV vaccination^26^. While these observations appear to contrast our results, our study was different: all volunteers in our study received a heterologous booster regimen consisting of an initial two doses of inactivated virus for priming followed by DNA- or mRNA-vaccines for a further 2 or 3 doses. There did not appear any differences in neutralization activity after either repeating mRNA- or combining DNA-/mRNA boosters (Fig. 1C,D). However, our results cannot exclude the effect of time on the neutralizing activity of antibodies due to differences in the timings of sample collection after 4 and 5 booster doses. This disparity precludes a direct comparison of vaccine effectiveness between the two groups. Additionally, our study employed the surrogate SARS-CoV-2 neutralization test (sVNT) to assess neutralizing antibodies, a method known to yield varied results compared to other techniques.

Our previously-published data from the same participants who initially received 2 doses of CV vaccination revealed a median value of IFN-γ release of 0.06 IU/mL (IQR at 0.02–0.15 IU/mL) after in vitro stimulation with RBD or 0.10 IU/mL (IQR at 0.03–0.32 IU/mL) after stimulation with S1S2 antigens^20^. The present study followed up these participants after receiving either their 4th or 5th doses of vaccine and we showed increased levels of IFN-γ production after stimulation with either RBD protein or S1S2 antigens from boosted participants compared to vaccination with two doses CV (*p* < 0.0001, Supplementary Fig. S8). The SARS-CoV-2 antigens that we used in the IFN-γ releasing assay comprised the RBD epitope that is specific for CD4+ T cells or the S1S2 subunits of the spike protein that can activate both CD4+ and CD8+ T cells^30^. Our observations are in agreement with a previous publication which showed that T cell responses via IFN-γ release were enhanced after the second booster dose^31^. A study of heterologous vaccination with two doses of DNA vaccine followed by mRNA vaccine as a booster reported that 92.8% of the participants were positive for IFN-γ release^32^. We did not detect any significant differences in surrogate neutralizing activity (see above) or IFN-γ after either repeating mRNA- or combining DNA-/mRNA-boosters, but it is reported that even though the anti-RBD IgG levels after homologous or heterologous vaccination were similar, the IFN-γ responses after heterologous vaccination were found to be higher than after homologous vaccination^24^.

Memory T cell responses in 11 of 12 participants after heterologous DNA vaccination followed by boosting with mRNA vaccine were increased in both CD4+ and CD8+ T cells^33^. A study of memory T cell phenotypes after first vaccination showed increased numbers of EM and TEMRA CD4+ or CD8+ T cells but these were lower after the second and the third vaccination^34^. Our study is the first observation in a Thai heterologous booster vaccination program which investigates changes in memory T cell subpopulations upon in vitro stimulation of PBMC with Omicron peptide, compared to unstimulated circulating cells. Our results showed an increased trend of naïve and CM phenotypes of both CD4+ and CD8+ T cells after in vitro stimulation, which suggests that exposure to Omicron antigens will drive T cells into a lymphoid resident T cell phenotype. Those cells may home to lymphoid organs for enhanced immunity against SARS-CoV-2, whereas effector memory cells function as blood and tissue surveillance cells^35,36^.

In conclusion, this study shows high surrogate neutralizing antibody levels after 4 or 5 doses of vaccination, but no significant differences following either repeating mRNA- or combining DNA-/mRNA-boosters. T cells from participants after 4 or 5 doses had increased IFN-γ production after in vitro stimulation with SARS-CoV-2 S1S2 peptides indicating enhanced CD8+ memory cell function after this additional vaccination from 2 doses of CV. This study has several limitations including a small sample size, particularly low numbers of participants in some of the heterologous booster combinations because participants being followed up from our previous study. Additionally, there were unequal time intervals between booster administration and sampling. Further follow up studies are required to determine if this enhanced immunity results in more protection against SARS-CoV-2 infection and prevents development of severe disease. This will be important to evaluate the cost effectiveness of continued booster programs for the general population using either mRNA- or viral vector vaccines^37^. SARS-CoV-2 infection is more problematic in people with chronic underlying conditions, including the elderly as well as immunocompromised individuals^38^ who are at high risk of developing severe disease from SARS-CoV-2 and should receive a regular boosting dose to maximize protection^39,40^. Previous reports of severity and mortality of COVID-19 infected Thai patients who received heterologous vaccination showed no severe infection after the fourth dose, with highest protection reported at 14–90 days after third dose vaccination^40^. These reports and our new data lead us to propose that the booster vaccination should continue to maximize effectiveness of immunity especially for people at high risk of developing severe disease following infection. Our data also support the conclusion that the number of doses of vaccination are important for maintaining immunity^25^.

### Supplementary Information

Supplementary Information.

## Data Availability

All data in this article are available in the paper or the Supplementary Material.
