# Supplementary material for: A two-arm analysis of the immune response to heterologous boosting of inactivated SARS-CoV-2 vaccines
Source: Sci Rep. 2023 Oct 31;13:18762. doi: 10.1038/s41598-023-46053-8 (PMC10618206; doi:10.1038/s41598-023-46053-8)
Supplement: Supplementary file 1 — Supplementary Information. [file 41598_2023_46053_MOESM1_ESM.pdf]

Supportive Information

A Two-Arm Analysis of the Immune Response to Heterologous Boosting of Inactivated SARS-CoV-2 Vaccines among Thai Individuals

Arnone Nithichanon[1,2], Ludthawun Kamuthachad[1], Kanin Salao[1,2], Wisitsak Phoksawat[1,2], Chatcharin Kamsom[1], Surasakdi Wongratanacheewin[1], Chonlatip Pipattanaboon[1], Sakawrat Kanthawong[1], Umaporn Yordpratum[1], Sirinart Aromseree[1], Atibordee Meesing[4], Piroon Mootsikapun[4], Steven W. Edwards[1,3], Supranee Phanthanawiboon[1,*]

Affiliation

[1] Department of Microbiology, Faculty of Medicine, Khon Kaen University, Khon Kaen, Thailand

[2] Research and Diagnostic Center for Emerging Infectious Diseases (RCEID), Khon Kaen University, Khon Kaen, Thailand

[3] Institute of Infection, Veterinary and Ecological Sciences, University of Liverpool, United Kingdom

[4] Infectious Disease Unit, Department of Medicine, Faculty of Medicine, Khon Kaen University, Thailand

*** Correspondence:**

Supranee Phanthanawiboon

E-mail: supraph@kku.ac.th

Supplementary Table S1. Days after the blood collection post each dose.

Parameter	4 Doses (N = 40)	5 Doses (N = 16)	p-value
**Days after the blood collection post each dose, median (interquartile range)**			
1st dose	451 (435 - 480)	480 (468 - 482)	0.0084*
2nd dose	417 (406 - 452)	451 (440 - 453)	0.0227*
3rd dose	360 (356 - 374)	365 (357 - 377)	0.9962
4th dose	217 (204 - 219)	217 (216 - 226)	0.1802
5th dose	N/A	19 (8 - 39)	N/A

*, statistically significance, N/A, not available

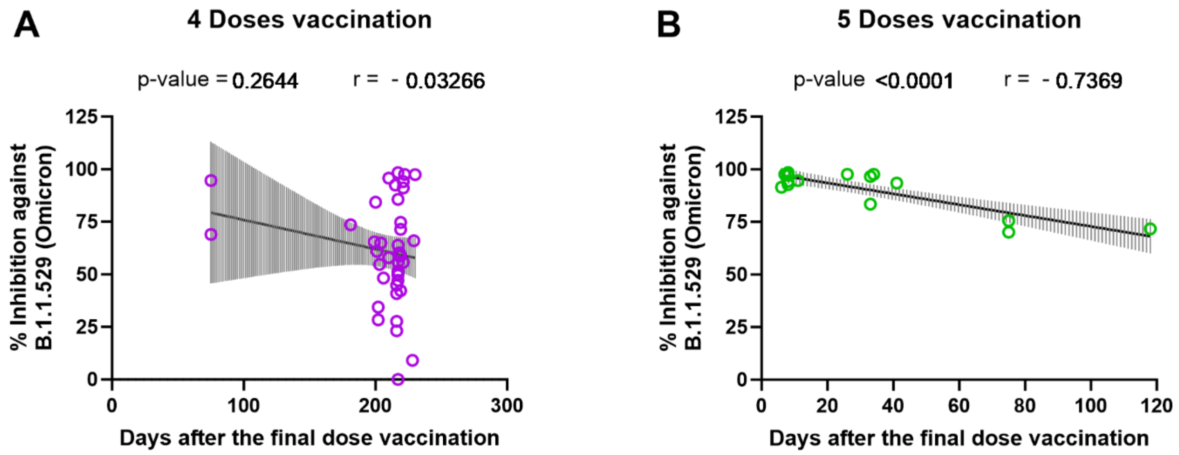

Supplementary Figure S1. Correlation between blood collection time after last vaccination dose and % inhibition of Omicron. The % inhibition of B1.1.529 (Omicron) by serum from participants that received 4 doses **(A)** or 5 doses **(B)** were plotted against the time of blood collection after the final vaccination and analysed by linear regression. Statistical significance was determined at a p-value < 0.05.

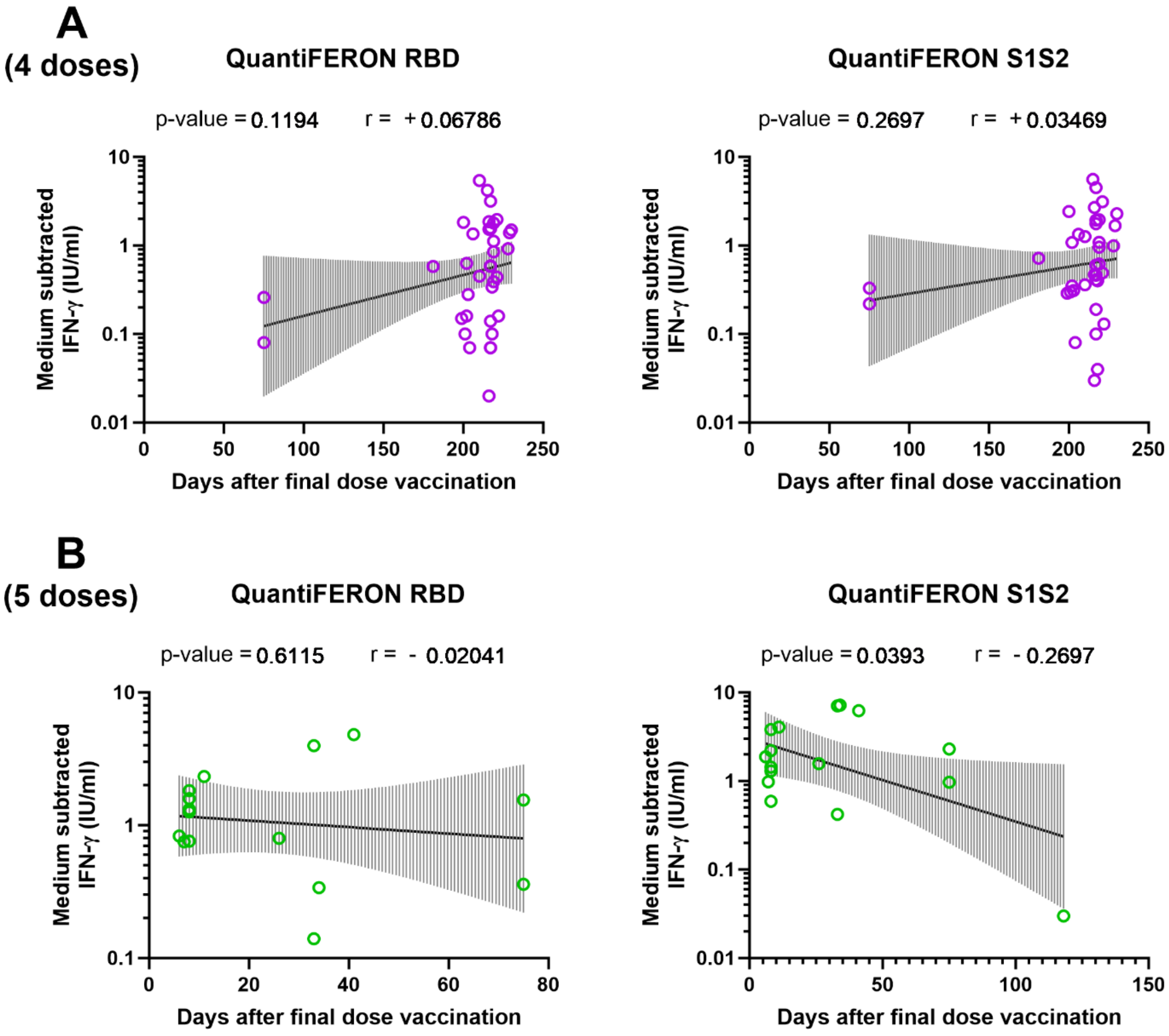

Supplementary Figure S2. Correlation between blood collection time after last vaccination dose and IFN-γ levels. IFN-γ levels from samples after 4 doses **(A)** or 5 doses group **(B)** were plotted against time of blood sampling after the final vaccination and anlaysed by linear regression. Statistical significance was determined at a p-value < 0.05.

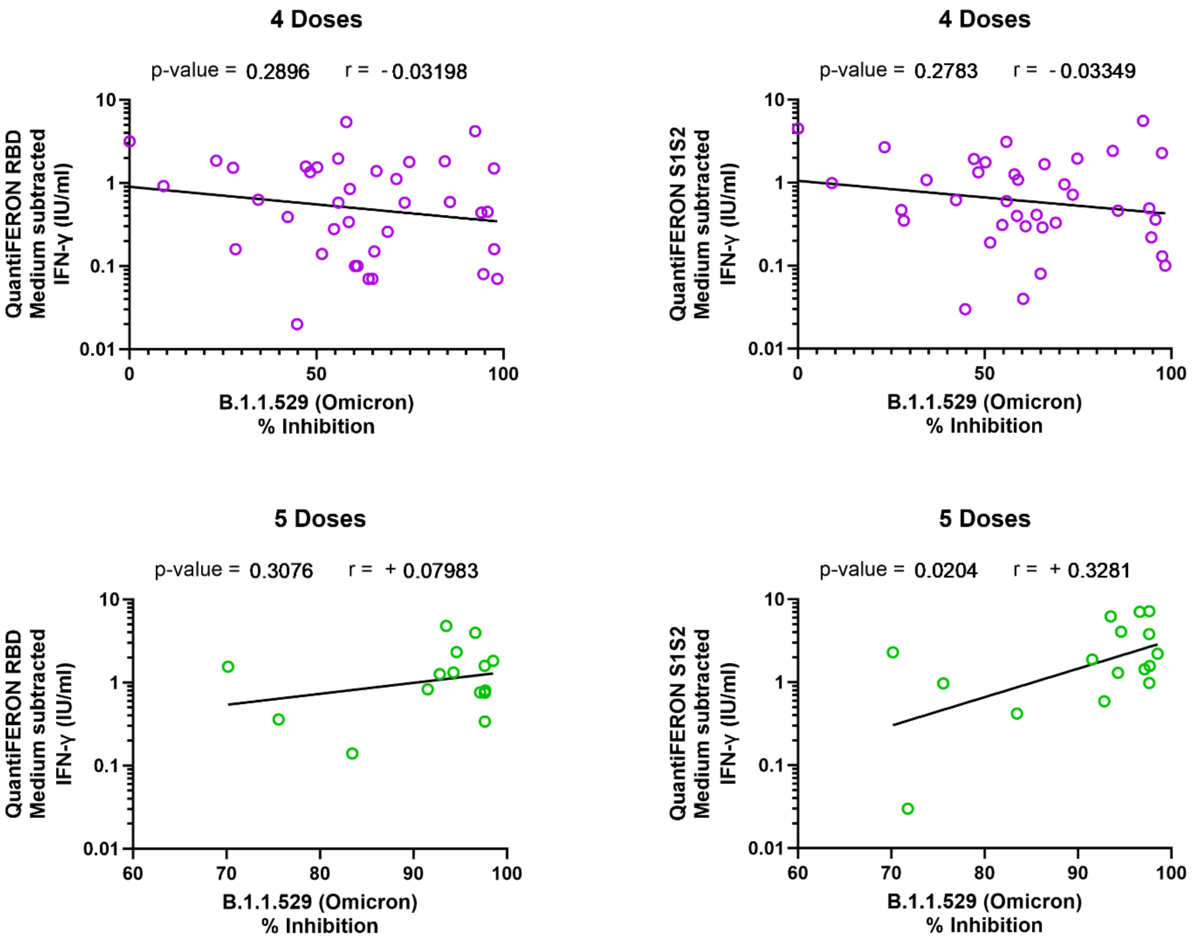

Supplementary Figure S3. Correlation of IFN-γ release and % inhibition capacity of serum against Omicron. Correlations of Log_{10} transformed IFN-γ levels stimulated by RBD or S1S2 peptides and % inhibition of serum against Omicron from participants after 4 doses (○) or 5 doses (○) group were analysed with linear regression. Statistical significance was determined at a p-value < 0.05.

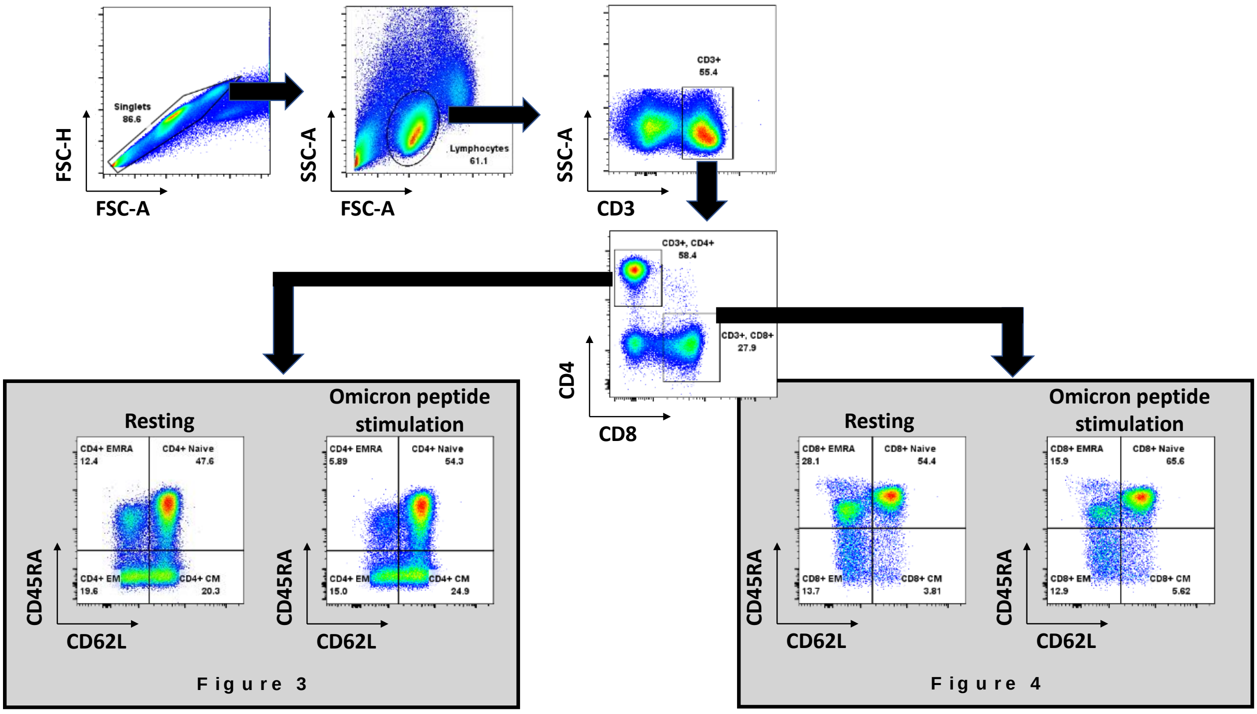

Supplementary Figure S4. Flow cytometry gating strategies for memory T cell phenotypes. PBMCs from participants were cultured with medium (control, Resting) or Omicron peptide prior surface marker staining and flow cytometry analysis. Singlet cells were gated according to forward scatter (FSC-H) and forward scatter (FSC-A). Lymphocyte populations were initially identified according to side scatter area (SSC-A) and FSC-A. T cells were gated after staining as CD3+. Memory helper T cells were CD4+ positive while memory cytotoxic T cells were CD8+ positive. After gating of CD4 and CD8 cells, memory phenotypes were sub-grouped as: naïve (CD45RA+ and CD62L+); central memory (CM; CD45RA- and CD62L+); effector memory (EM, CD45RA- and CD62L-); effector memory RA (EMRA, CD45RA+ and CD62L-).

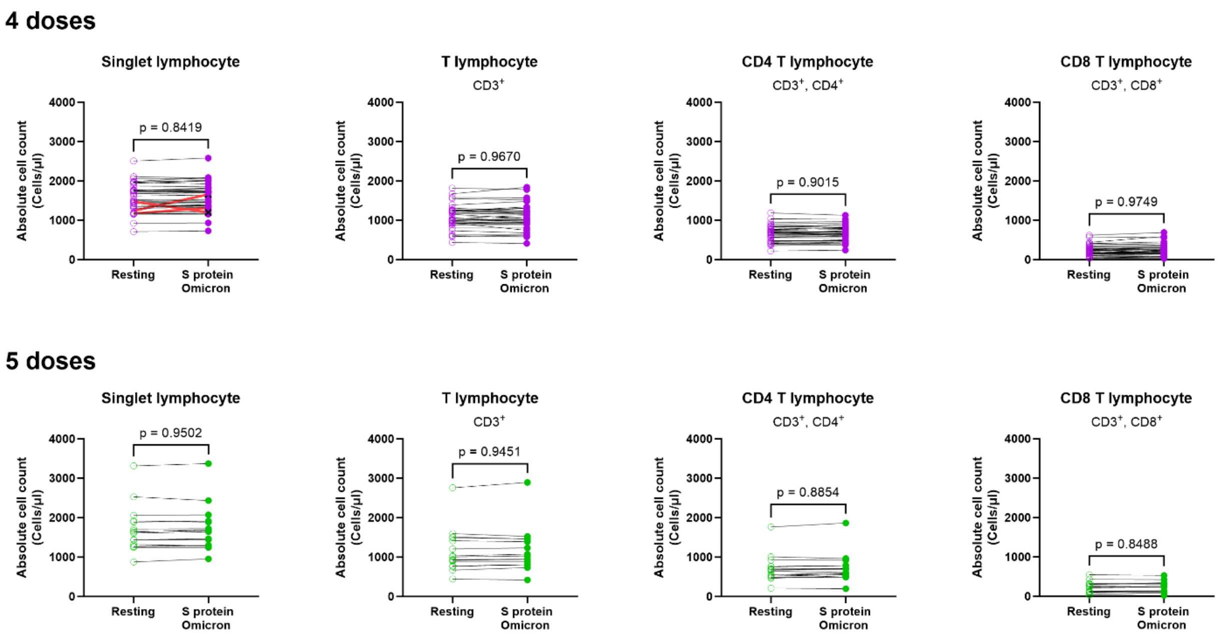

Supplementary Figure S5. Comparison of absolute numbers of lymphocytes and T lymphocyte subsets after stimulation with Omicron peptide. Sample with changes in singlet cells of >10% were excluded from data analysis (red labeled lines)

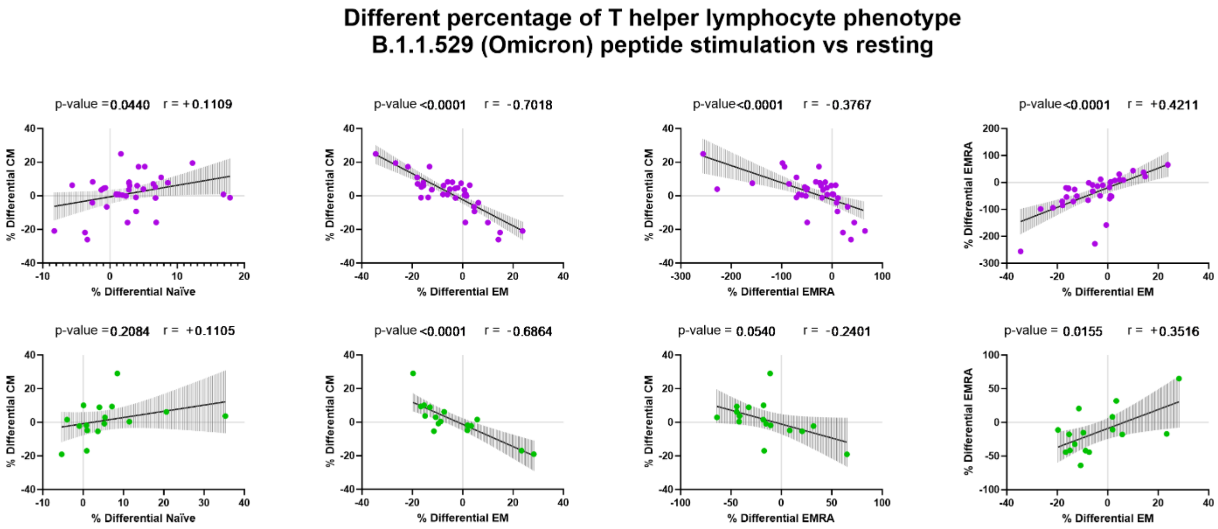

Supplementary Figure S6. Correlation of differential T helper lymphocyte phenotypic pairs after Omicron peptide stimulation. Differences in percentages of each T helper lymphocyte phenotype were calculated and compared with absolute cell number after peptide stimulation and in unstimulated resting cells. Correlations were performed by linear regression. Data of participants receiving 4 doses (●) or 5 Doses (●) are shown as dot plots with regression line. Statistical significance was determined at a p-value < 0.05.

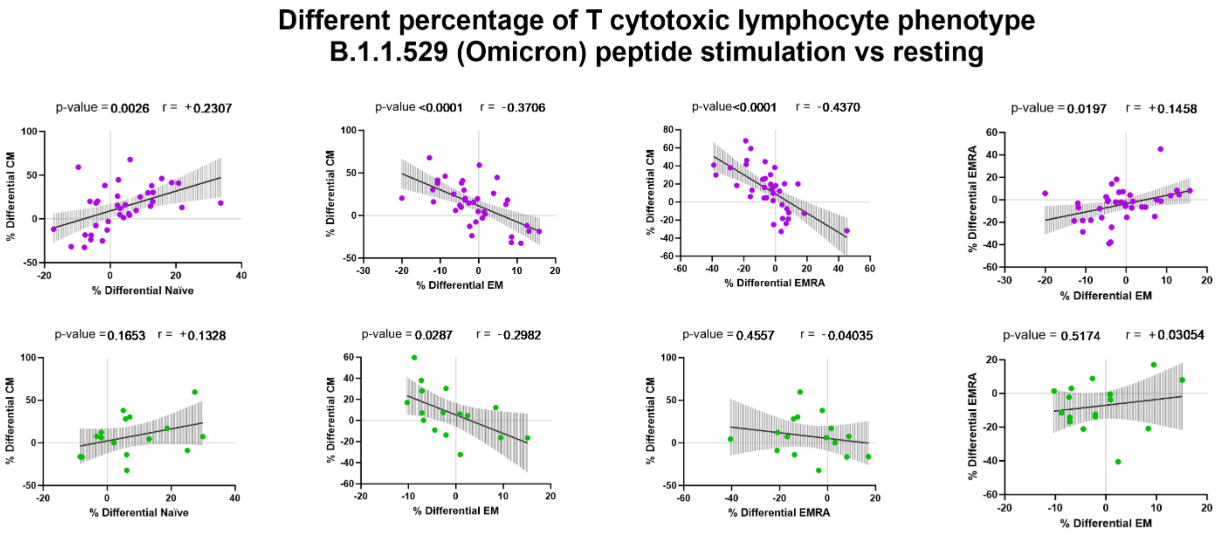

Supplementary Figure S7. Correlation of differential cytotoxic T lymphocyte phenotypic pairs after Omicron peptide stimulation. Differences in percentages of each cytotoxic T lymphocyte phenotype were calculated and compared with absolute cell number after peptide stimulation and in unstimulated resting cells. Correlations were performed by linear regression. Data of participants receiving 4 doses (●) or 5 Doses (●) are shown as dot plots with regression line. Statistical significance was determined at a p-value < 0.05.

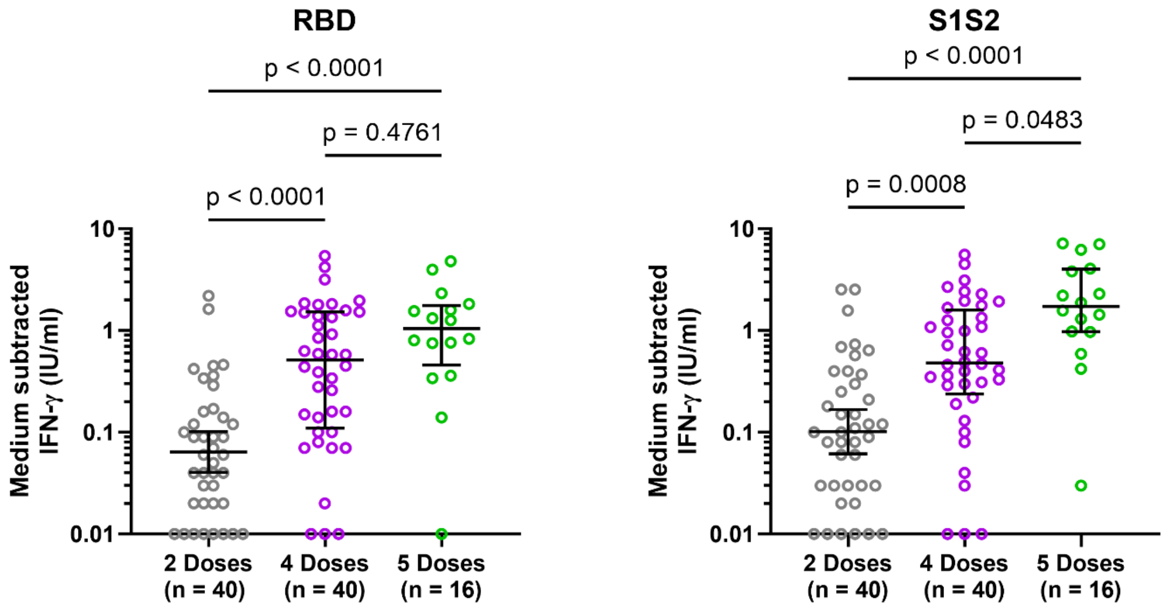

Supplementary Figure S8. Comparison of specific T cell responses measured as IFN-γ release upon *in vitro* stimulation with SARS-CoV2 receptor-binding domain (RBD) or S1S2 peptides after two or four or five doses of vaccination. Peripheral blood mononuclear cells (PBMCs) from participants after 4 doses or 5 doses were stimulated with RBD or S1S2 peptides. IFN-γ release was quantified by using QuantiFERON. Data are shown as individual dot plot after medium (control) subtracted IFN-γ levels (○; open grey circle for 2 doses, ○; open purple circle for 4 doses, ○; open green circle for 5 doses) with horizontal lines showing median and interquartile ranges. IFN-γ levels release by samples after 2 doses or 4 doses or 5 doses in response to RBD or S1S2 peptides were compared using Kruskal-Wallis test with Dunn's multiple comparisons test. Statistical significance was determined at a p-value < 0.05.
